# An Early Presentation of Buried Bumper Syndrome

**DOI:** 10.7759/cureus.10969

**Published:** 2020-10-15

**Authors:** Mohamad F Ayas, Gilles J Hoilat, Saif Affas

**Affiliations:** 1 Internal Medicine, Ascension St. John Hospital and Medical Center, Detroit, USA; 2 Internal Medicine, State University of New York Upstate Medical University, Syracuse, USA

**Keywords:** buried bumper syndrome, percutaneous endoscopic gastrostomy

## Abstract

Percutaneous endoscopic gastrostomy (PEG) is a well-established and successful method of nutritional delivery. Complications, although rare, are divided into early or late. Buried bumper syndrome (BBS) is usually a late complication of PEG tube insertion and can cause many issues such as pressure necrosis, peritonitis, and septic shock. Endoscopic evaluation is the definitive diagnosis, and treatment depends on each patient and the degree of depth of disc migration. We present to you a case of buried bumper syndrome in a 66-year-old female that was initially thought to be complicated with peritonitis, and surprisingly occurring only one week after initial PEG tube placement.

## Introduction

Percutaneous endoscopic gastrostomy (PEG) is a widely used method of nutritional delivery in patients who require long time enteral nutrition [1,2], such as patients with neurologic deficits, dysphagia, head and neck cancers, major traumas, burns, and short-bowel syndrome [3], as gastric feeding is the most common type of enteral feeding [1]. PEG tube placement was first introduced in 1980 by Gauderer and Ponsky [4], and was found to be a better alternative than other surgical methods for feeding tube placements, given its low cost and rarely needing general anesthesia [1]. Complication rates vary and are divided into minor and major complications, and many classify it as early or late [1]. Minor/early complications are three times more likely to happen and include wound infection, bleeding risk within and around the peritoneal area, abdominal organ perforation, tumor seeding, stomal infection, and possible aspiration during the procedure [5]. As for major/late complications, they mainly include necrotizing fasciitis, gastro-cutaneous fistulas, and buried bumper syndrome (BBS), with or without peritonitis [1,5]. BBS is a rare complication of PEG tube placement and is defined when the inner bumper migrates through the gastric wall and is lodged between the gastric wall and skin [3]. Although considered a late complication, it can present early on in rare situations, as in our case, and is manifested with abdominal pain, tube malfunction, and leakage around the tube [3].

## Case presentation

**Figure 1:**
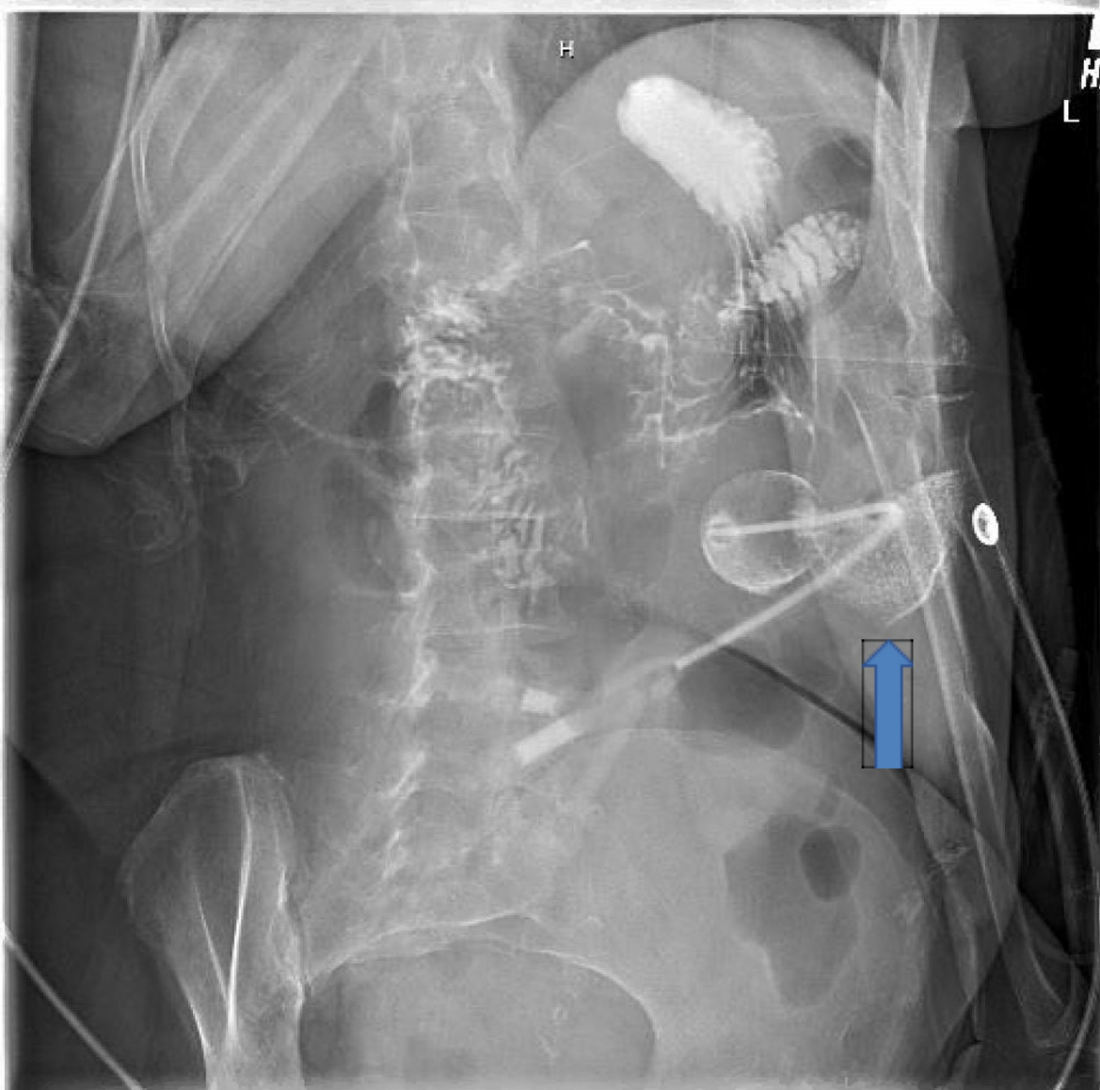
Gastrostomy X-ray With Gastrografin Injection Through PEG Tube Showing Extravasation at the Skin Surface (Blue Arrow)

A 66-year-old female with a past medical history significant for cerebral vascular accident (CVA) with right-sided deficits, aphasia, and hypertension was transferred from an extended care facility for PEG tube malfunction. The patient was initially admitted seven days prior, and in that time, had a PEG tube placed for long-term enteral nutrition. During readmission, the patient was found to be septic with a heart rate of 123 beats per minute and a low-grade fever of 99.3°F; the rest of vital signs were within normal limits. Laboratory findings showed a white blood cell count of 21.81 k/mcl, a platelet count of 556 k/mcl with a normal lactic acid of 1.8 mmol/L, and two blood cultures were obtained. Physical exam showed minimal grimacing of the patient upon palpation of the abdomen around the PEG tube and minimal redness and swelling around the PEG tube. Peritonitis versus local stomal infection was suspected, so the patient was started on intravenous (IV) cefepime and metronidazole. Further studies such as gastrostomy X-ray with Gastrografin injection through PEG tube was performed and showed that the gastrostomy bolster had been retracted along the tube tract, with extravasation at the skin site only without evidence of a peritoneal leak (Figure 1). A gastroenterology consult was then placed to evaluate for endoscopic repair. The patient underwent endoscopy, which showed a normal esophagus and stomach with no evidence of an internal PEG tube balloon, consistent with buried bumper syndrome. The old PEG tube was pulled all the way out, and the patient underwent a successful endoscopic PEG tube replacement. The patient had significant clinical improvement, and laboratory findings normalized. Blood cultures were negative, and antibiotics were discontinued on day four without clinical deterioration.

## Discussion

PEG tubes were initially introduced in the 1980s by Gauderer et al., and although considered safe, this procedure, like all others, can present with complications [4,6]. Minor/early complications are usually described as those that occur in <30 days. However, major/late complications are those that take months or years to develop. The buried bumper syndrome was first described in 1988 [7,8] and is considered a late complication of PEG tube placement. A PubMed search using "early buried bumper syndrome" was done, and 25 articles appeared from 2000 to 2020. We excluded 15 articles: three were not in English, and the other 12 articles did not have BBS as an early presentation. Thus, leaving us with only 10 cases of BBS reporting its occurrence within the first month, as in our case. BBS is described as when the internal stump of the probe migrates and is located anywhere between the gastric wall and the skin [3]. It usually develops as a consequence of the tight positioning of the external bumper of the PEG tube against the abdominal wall [5]. This can also occur when the PEG tube is strongly pulled away from the person, moving the internal bumper into the gastrostomy tract [5]. Other etiologies occur when there is an increase in hydrochloric acid, which produces physical alterations in the internal bumper, when the patient is obese or has a chronic cough, as well as inadequate gastrostomy tube material or size [5]. It has an incidence of 0.3%-2.4%, and the classic sign of BBS is the inability to advance the PEG tube further [5,9,10]. Other findings include the inability to insert feedings, loss of patency, or leakage around the PEG tube [1]. It was noticed that immunocompromised patients or those with a low body mass index (< 20) are at increased risk of developing BBS [9]. Diagnosis can be made clinically by history and physical examination; however, definitive diagnosis is needed and achieved mainly by endoscopy. Abdominal ultrasonography (USG), fluoroscopy, and computed tomography (CT) scan can also aid in the diagnosis [11]. In our case, fluoroscopy was done and showed leakage around the skin site. However, one must always remember that negative fluoroscopy does not rule out BBS, as the PEG tube will still show contrast material entering the stomach because the gastrostomy tract is still open [5]. There are a few BBS classifications; however, the one described by Orsi et al. is the most widely used, and it is based on symptomatology and migration of the internal stump and is divided into three grades [5,12]:

Grade I: partial migration with asymptomatic presentation or mild symptoms such as abdominal pain or ostomy infection; 

Grade II: subtotal migration, in which the patient presents with dysfunction of the tube and extravasation of the nutrition; and 

Grade III: total migration that is manifested by tube obstruction.

Currently, there are no guidelines on which we could base the treatment; however, many follow the grading system mentioned above. Grade I normally undergo endoscopic management, but for Grade II, or III, a surgical approach is preferred [5]. In general, removing the PEG tube is the most widely described treatment for BBS, either endoscopically or surgically [7,11]. It was found that in the acute setting of BBS, conservative and endoscopic approach cases are usually not suitable as the stoma tract is immature, but the treatment depends on the patient and depth of migration of the disc [11]. In our case, the patient was successfully treated with endoscopic PEG tube removal and replacement. All in all, BBS can be avoided by checking the external bolster of the gastrostomy tube and leaving it approximately 0.5 cm to 1 cm from the abdominal wall to prevent excessive traction, as well as receiving daily care in which the gastrostomy tube itself should be pushed in and out 1 cm to 2 cm and rotated 360 degrees, with periodic measurements of the external PEG tube [5]. Therefore, early recognition of BBS is essential for the prompt and proper management of affected patients. However, guidelines are yet to be established, and further clinical data is needed for optimal management of BBS.

## Conclusions

BBS is most commonly a late complication of PEG tube insertion. However, one must always keep in mind that this complication, although rare, can occur early in the course, as we presented, and can manifest as abdominal pain, leakage around the tube site, or even tube malfunctioning. Although BBS can be identified through history and physical exam, imaging such as a CT scan, USG, or fluoroscopy may also aid in diagnosis and early detection. BBS is usually managed conservatively, endoscopically, or surgically. Moreover, daily care of the gastrostomy tube, such as measurements of the protruding external portion of the PEG tube, is necessary to recognize and treat early migration.
